# Why do academicians share knowledge? A study of higher education institutions in India

**DOI:** 10.3389/fpsyg.2023.1181030

**Published:** 2023-09-01

**Authors:** Asad Ahmad, Md Sarwar Alam, Mohd Danish Kirmani, Dag Øivind Madsen

**Affiliations:** ^1^Department of Management, Jamia Hamdard, New Dehli, India; ^2^Department of Business Administration, Aligarh Muslim University Murshidabad Centre, Murshidabad, West Bengal, India; ^3^Department of Management, SRM University AP, Amaravati, India; ^4^USN School of Business, University of South-Eastern Norway, Kongsberg, Norway

**Keywords:** knowledge sharing, attitude to share knowledge, intention to share knowledge, actual knowledge sharing behavior, scale development, behavioral intentions

## Abstract

**Purpose:**

Indian higher education institutions are diverse in nature; there are institutions with good infrastructure and resources as well as institutes that have little in terms of resources and infrastructure. Keeping in mind the relevance of knowledge sharing in academic institutions, the researchers in the present study have tried to find factors determining the knowledge sharing behavior of the academicians of different institutes in India.

**Design:**

The researchers in the present work have expanded on extant research by demarcating factors that affect the knowledge sharing behavior of academicians. A structured questionnaire was shared through e-mail and social media groups, and a snowball approach was used to reach out to the maximum number of respondents.

**Findings:**

The present study offers an integrated and extended theory of planned behavior (TPB) theoretical model, augmenting it with constructs such as motivation and the opportunity to share knowledge adapted from related studies. The findings of this research provide theoretical as well as practical suggestions in determining and explaining the knowledge sharing behavior of academicians.

**Originality:**

The researchers in the present study have tried to present a shorter and more reliable scale that can be used to assess the behavioral intentions of academicians to share knowledge.

## 1. Introduction

The present business world is full of competition and uncertainties. It has become challenging for any business or organization to survive and grow in their respective sectors. The world economy now relies on knowledge and it has been recognized as a strategic resource for sustainable competitive advantage (Howell and Annansingh, 2013; Islam et al., 2015; Lee, 2016; Santoro et al., 2018). In the contemporary knowledge-intensive age, the effective flow of knowledge resources has become an important criterion for any organization to achieve a long-lasting competitive advantage (Obrenovic et al., 2021; Kumar et al., 2022). Knowledge sharing has become a critical factor for achieving such a competitive edge (Obrenovic et al., 2021). Organizations across different sectors have comprehended the benefits and rewards of knowledge sharing practices. Such practices have helped them to achieve their goals (Al-Kurdi et al., 2020). Like these organizations, knowledge sharing plays a key role in educational institutions (Jameel et al., 2021). Specifically, higher education institutions (HEIs) are like knowledge control rooms that have a vital role in the socio-economic advancement of nations (Syed et al., 2021; Shaukat et al., 2023). Knowledge management and knowledge sharing are key emerging issues in such institutions (Al-Kurdi et al., 2020). Furthermore, with the internationalization of institutions and the development of knowledge societies across the world, there is now increased pressure on HEIs to develop a positive environment for knowledge sharing on their premises (Chedid et al., 2022). It has become vital for universities to establish knowledge management systems and promote knowledge sharing within their institutions (Al-Kurdi et al., 2018).

Moreover, it has been suggested that the individuals, i.e., the employees, are the key facilitators of knowledge management and knowledge sharing activities in an organization (Prabhakar et al., 2018; Obrenovic et al., 2020). Individuals with different characteristics bring a wide set of knowledge, skills, and abilities, and have the potential to develop and modify the existing knowledge of an organization (Esmaeelinezhad and Afrazeh, 2018). In the case of HEIs, the concept of knowledge sharing has been adopted by academicians over the last decade (Al-Kurdi et al., 2018). Historically, academicians have practiced freedom and autonomy in their institutions (Sporn, 1996). They are typically recognized as the intellectual leaders of society who play a major role in creating and utilizing knowledge and intellectual property through research, teaching, and learning (Kim and Ju, 2008; Al-Kurdi et al., 2020; Jameel et al., 2021). Additionally, their performance and productivity are the key determinants of the ranking of universities, which include publications, conference participations, community services, and professional activities (Massoudi et al., 2020; Syed et al., 2021). All such activities cannot be empowered without knowledge sharing practices (Akosile and Olatokun, 2020; Shaukat et al., 2023). It is not possible for faculty members to accomplish such responsibilities without collaborations and partnerships in form of knowledge sharing (Mutahar et al., 2022). On the other hand, oftentimes, such individuals may give priority to their individual goals (Kim and Ju, 2008) and may fear losing their knowledge power by sharing their knowledge with fellow members (Al-Kurdi et al., 2018). Such unwillingness to share knowledge could weaken the institution’s efforts to attain its goals, foster research collaborations, and boost innovation in society at large (Al-Kurdi et al., 2020). Therefore, identifying and comprehending the factors shaping an individual’s knowledge sharing behavior is of prime importance in the case of knowledge management professionals. In the case of academicians, it is essential to examine the attitudes, actions, and behaviors related to knowledge sharing in academic environments (Shaukat et al., 2023). It is equally important to understand what factors can affect the intentions to share knowledge, which in turn could enhance universities’ knowledge management and knowledge sharing practices and bring innovation (Al-Kurdi et al., 2020). Various studies have suggested that when it comes to knowledge sharing practices, an individual’s behavior coming out of his/her own personality as well as the surrounding conditions and other factors play a key role (Ajzen, 1991; Cheng et al., 2013; Razak et al., 2016; Safa and Solms, 2016). Therefore, in line with the imperativeness of knowledge sharing practices in HEIs and an individual’s behavioral approach toward knowledge sharing coming out of personal factors and the surrounding conditions, the present study is an attempt to understand the knowledge sharing approach and pattern of academicians, i.e., the university teachers working in the HEIs of India.

## 2. Literature review

### 2.1. Knowledge management

In the knowledge-driven global economy, knowledge management has been found to be a key element in developing a sustainable competitive advantage (Santoro et al., 2018). Davenport and Prusak (1998) defined knowledge management as “the process of capturing, storing, sharing, and using knowledge.” It involves people who are responsible for carrying out all these activities. Zhang et al. (2015) defined it as “certain organizational approaches to achieve organizational goals through the effective use of knowledge.” García-Sánchez et al. (2017), in their study, mentioned that the knowledge management concept is multidimensional. Some studies have considered it to consist of four processes, namely “acquisition or creation,” “storage or retrieval,” “sharing or transfer,” and “usage or application” (García-Sánchez et al., 2017; Esmaeelinezhad and Afrazeh, 2018). Some have considered it to be five-dimensional, encompassing processing related to capturing, integrating, sharing, using, and maintaining knowledge (Farnese et al., 2019), whereas some have argued for three dimensions, namely, knowledge acquisition, knowledge sharing, and knowledge utilization (Tiwana, 2002). Among all the processes and activities of knowledge management, knowledge sharing has been considered to be the key process and has received immense attention from academic researchers and industry practitioners (Witherspoon et al., 2013; Islam et al., 2015; Razak et al., 2016; Al-Kurdi et al., 2018; Prabhakar et al., 2018; Obrenovic et al., 2020). Abubakar et al. (2019) cited knowledge sharing as a critical factor of the knowledge management process. They mentioned that knowledge sharing among peers in the form of collaboration enables the knowledge creation process by providing a competitive advantage to the organization over its competitors.

### 2.2. Knowledge sharing

According to Razak et al. (2016), knowledge sharing is the act of exchanging and disseminating ideas, experiences, and knowledge with others so that it can be used, retained, and sustained in an organization. Doğan and Doğan (2020) defined it as “the transfer or dissemination of information from one person, group, and organization to another person, group, and organization.” Lin (2007) defined knowledge sharing as “a social interaction culture, involving the exchange of employee knowledge, experiences, and skills through the whole department or organization.” Furthermore, Ortiz et al. (2017) defined it as “the activity of sharing individuals’ experience and professional knowledge with others within teams/organizations to help them learn new ideas.” It involves the dissemination of organizational knowledge among the employees in order to get them involved in focused actions and innovation within the organization (Islam et al., 2015). Witherspoon et al. (2013) considered it to be an important process for developing and sustaining new and innovative business processes.

### 2.3. Research gap

Previous studies have shown that knowledge sharing has been widely examined for professional and non-professional groups around the world belonging to different sectors and work environments (Shaukat et al., 2023). Various organizations across other sectors have leveraged the benefits and advantages of knowledge sharing practices. They have conducted research on knowledge sharing so that organizational goals could be achieved effectively using such practices (Al-Kurdi et al., 2020). Kumar et al. (2022) identified that knowledge sharing practices are being executed differently as per the industry or sector the organization belongs to. Some of the key sectors where knowledge sharing research has taken place worldwide include tourism, healthcare, information technology (IT), banking, insurance, and e-commerce (Lee and Hong, 2014; Liou et al., 2016; Safa and Solms, 2016; Mafabi et al., 2017; Park and Kim, 2018; Obrenovic et al., 2020; Rohman et al., 2020; Kumar et al., 2022). Le and Nguyen (2023) studied employees’ knowledge sharing behavior in Vietnamese firms. Kumar et al. (2022) investigated the knowledge sharing behavior of the employees belonging to the hotel industry in the travel destinations of India. Mohd Rasdi and Tangaraja (2022) studied knowledge sharing behavior among Malaysian public service administrators. Obrenovic et al. (2020) conducted an empirical study to examine the knowledge sharing behavior among the employees of various technological firms in Croatia in industries such as electronics, medicine, IT, and biochemistry. Furthermore, the last decade has witnessed some imperative studies on knowledge sharing behavior in the education sector, especially HEIs (Sadiq Sohail and Daud, 2009; Al-Kurdi et al., 2018, 2020; Prabhakar et al., 2018; Chedid et al., 2019, 2022; Farrukh et al., 2020; Jameel et al., 2021; Syed et al., 2021; Mutahar et al., 2022; Kaba et al., 2023; Shaukat et al., 2023). Kaba et al. (2023) examined the demographic characteristics differences in the knowledge sharing behavior of non-academic staff of two universities in India and the UAE. Shaukat et al. (2023) studied the impact of personality traits on the knowledge sharing behavior of academicians belonging to public universities in Pakistan. Mutahar et al. (2022) investigated the impact of trust on academician’s knowledge sharing behavior in Malaysian research universities. Finally, Chedid et al. (2022) examined the relationship between individual factors and attitude toward knowledge sharing among professors and researchers. Table 1 shows a summary of some of the recent key studies conducted in the area of HEIs.

**Table 1:** Recent studies conducted in the area of HEIs.

Author and Year	Region	Objectives	Constructs	Methodology	Results
Kaba et al. (2023)	India and UAE	Demographic characteristics differences in knowledge sharing behavior of non-academic staff.	Attitude, subjective norms, behavioral intention, and knowledge sharing behavior	Empirical study of 467 non-academic staff members from two academic universities in India and the UAE.	There were significant geographical location differences in attitude, behavioral intention, and knowledge sharing behavior.
Shaukat et al. (2023)	Pakistan	The impact of personality traits on the knowledge sharing behavior of academicians in the public sector.	Five personality traits, knowledge sharing behavior, written contributions, organizational communications, personal interactions, and communities of practice	Empirical study of 237 academicians in a public university of Pakistan.	The personality trait openness to experience had a significant and positive impact on KSB The personality traits extraversion and agreeableness positively predicted KSB.
Mutahar et al. (2022)	Malaysia	The effect of trust on academics’ knowledge sharing in Malaysian research universities.	Trust, organizational citizenship behavior, and knowledge sharing	Empirical study of 380 academicians in five Malaysian research institutions.	Trust has a positive and significant relationship with knowledge sharing.
Chedid et al. (2022)	Portugal	An examination of the relationship between individual factors and the attitude toward knowledge sharing among professors and researchers.	Intrinsic motivation, extrinsic motivation, social network, and attitude toward knowledge sharing	Empirical study of 176 professors and researchers in a public HEI.	Intrinsic motivation positively affects knowledge sharing attitude. Networking positively affects attitude in this institution.
Syed et al. (2021)	Pakistan	The interrelationship between knowledge sharing attitude and knowledge management processes along with the intervening role of subjective norms between KM processes and KS attitude.	Knowledge sharing attitude, subjective norms, knowledge sharing, and perceived behavioral control	Empirical study of 320 academic and 43 administrative staff members from research-based HEIs in Pakistan.	Perceived behavior control has a mediating impact on knowledge management processes and knowledge sharing attitude. Subjective norms have a mediating impact on knowledge management processes and knowledge sharing attitude.
Asghar and Naveed (2021)	Pakistan	The psychometric properties of the knowledge sharing behavior scale (KSBS) using academicians.	KSBS, written contributions, organizational communication, personal interaction, and communities of practice	Empirical study of 258 academicians at a university in Pakistan.	KSBS is not a valid measure for assessing knowledge sharing behavior in an academic context, specifically in the Pakistani environment.
Jameel et al. (2021)	Iraq	An examination of the effect of attitude, subjective norms, and perceived behavioral control on knowledge sharing among academic staff.	Attitude, subjective norms, perceived behavioral control, and knowledge sharing	Empirical study conducted among 163 academic staff members at three private universities.	There was a positive and significant impact of attitude, subjective norms, and perceived behavioral control on knowledge sharing among academic staff.
Farrukh et al. (2020)	Pakistan	An investigation into the relationship between individual characteristics and knowledge sharing in higher education institutes.	Extroversion, openness to experience, agreeableness, emotional intelligence, religiosity, neuroticism, and KS	Empirical study among 370 academic staff members of six HEIs of Pakistan.	Extroversion, openness to experience, agreeableness, emotional intelligence, and religiosity were positively associated with knowledge sharing, while neuroticism was found to be negatively associated with knowledge sharing.
Al-Kurdi et al. (2020)	UK	The role of organizational climate operationalized by organizational leadership and trust in academics’ knowledge sharing in HEIs.	Attitude, subjective norms, perceived behavioral control, intention, knowledge sharing behavior, leadership, trust, and organizational climate	Empirical study conducted on 257 faculty members of HEIs in UK.	Organizational climate has an exceptionally strong influence on academics’ knowledge sharing practices. Organizational leadership and trust has a positive relationship with academics’ knowledge sharing behavior.
Afshar Jalili and Ghaleh (2021)	-------	A summary of the application of the theory of planned behavior in predicting knowledge sharing behavior.	Attitude, subjective norms, perceived behavioral control, intention, and knowledge sharing behavior	Meta-analysis applied as a research methodology; 47 studies were included in this study.	Knowledge sharing behavior is determined jointly by knowledge sharing intention and perceived behavioral control.
Chedid et al. (2019)	Portugal	An examination of whether knowledge sharing intention has a positive relationship with collaborative behavior among professors and researchers in HEI.	Motivation, networking, organizational support, trust, subjective norms, attitude, intention, and collaborative behavior	176 professors and researchers from a public HEI in Portugal.	Intrinsic motivation and networking positively affect the attitude towards knowledge sharing. Trust strongly affects the knowledge sharing intention. Knowledge sharing intention has a positive influence on collaborative behavior.
Fauzi et al. (2019)	Malaysia	An investigation into Muslim academics’ knowledge sharing behavior and its relating predictors in the context of Malaysia.	Commitment, attitude, social networks, trust, management support, subjective norms, facilitating conditions, social media use, PBC, ISK, and KSB.	398 Muslim academics in Malaysia in 20 public and 5 private HLIs.	Social network, trust, management support, facilitating conditions, and social media are significant predictors of Muslim academics’ knowledge sharing behavior.
Al-Kurdi et al. (2018)	------	To provide a better understanding of knowledge sharing amongst academics in higher education institutions.	--------	Systematic literature review of 73 papers published in peer-reviewed journals over the last decade.	Individual factors: trust, personal attitude, motivation, affective commitment, subjective norms, personal expectation, and the relationship between knowledge and power. Organizational factors: organizational culture, climate, subcultures, reward systems, and management support.

Most such studies have used the theory of reasoned action (TRA) and the theory of planned behavior (TPB) models to understand the knowledge sharing pattern of academicians. The TPB is one of most powerful applied behavioral models but researchers have nevertheless anticipated the need to extend it further with some additional variables. Specifically, more variables should be included for understanding academicians’ behavior as primary knowledge sharing providers in HEIs (Al-Kurdi et al., 2020). According to Shaukat et al. (2023), knowledge sharing behavior should be treated as a multi-dimensional construct for an inclusive and vigorous understanding of such behavior and its sub-dimensions. The present study has identified a significant research gap in the form of two important variables missing from the discussions which are based on (i) motivations that push an individual to share knowledge, and (ii) opportunities that allow an individual to share knowledge. Al-Kurdi et al. (2020) mentioned in their discussion of limitations that motivation as a construct should be added to the research models to study academicians’ knowledge sharing behavior. The motivations of the academicians must be investigated for promoting such behavior and developing robust knowledge management strategies (Asghar and Naveed, 2021). As per the previous literature, universities have been facing a big challenge in form of academicians’ unwillingness to share knowledge with others (Mutahar et al., 2022). University managements need the motivation of these individuals for a positive approach toward knowledge sharing (Jameel et al., 2021; Obrenovic et al., 2021). Hence, it is critical to add motivation to share knowledge into the research model of further investigations. Furthermore, knowledge sharing can be expected from the academicians only in the presence of the availability of services and the right circumstances for sharing (Massoudi et al., 2020; Jameel et al., 2021; Karem et al., 2022). Academicians with a huge burden of work and assignments should be supported with favorable working conditions. This will lead them to a better state of mind for getting involved in knowledge sharing activities (Fauzi et al., 2019). Organizational support like facilitating conditions inspires knowledge sharing by stimulating employees’ willingness and innovative behavior (Kambey et al., 2018; Le and Lei, 2019; Obrenovic et al., 2020). Such factors can play a key role in enhancing the knowledge sharing behavior of university teachers (Javaid et al., 2020; Syed et al., 2021). Hence, opportunities to share knowledge is crucial to be examined in context of academicians’ knowledge sharing behavior in HEIs (Sadiq Sohail and Daud, 2009; Fauzi et al., 2019; Al-Kurdi et al., 2020).

Another important gap in the present research is the country or region where the study has been conducted. This is because the knowledge sharing behavior of the academicians is strongly associated with: (i) the characteristics of each country or region, and (ii) the culture of the institution based on various factors like culture, lifestyle, and beliefs of the knowledge workers (Al-Kurdi et al., 2018; Chedid et al., 2022; Farrukh et al., 2022). Cultural diversity often limits the generalizability of research (Al-Kurdi et al., 2020). The present study has been conducted in the Indian context where the results might be different from another nations. Moreover, to the best of the knowledge of the researchers, no study in India has been carried out with such an extensive number of variables to understand the knowledge sharing behavior of the academicians in Indian HEIs.

### 2.4. Objectives of the study

In line with the research gaps identified by the researchers, the study has the following objectives:

- To identify the key variables of knowledge sharing behavior among the academicians working in HEIs.
- To determine the relationships between the identified variables.
- To determine whether the academicians differ in exhibiting knowledge sharing behavior based on their age, gender, designation, total experience, and the type of university they are working with.
- To suggest a model based on the various relationships identified between the variables.

## 3. Research model and hypothesis development

Afshar Jalili and Ghaleh (2021) mentioned that researchers in the area of knowledge sharing are using socio-psychological theories to gain a better understanding of the psychological characteristics of an individual’s knowledge sharing behavior. According to Shaukat et al. (2023), universities should focus on how their academicians’ psychology works so that they can be helped to explore their potential for learning and research. When discussing the socio-psychological characteristics of an individual to understand their knowledge sharing behavior, the theory of planned behavior (TPB) has been widely used by knowledge sharing researchers (Al-Kurdi et al., 2020; Afshar Jalili and Ghaleh, 2021; Negara et al., 2021). Furthermore, in context of HEIs, this model has been given prominent place to understand the academicians’ knowledge sharing behavior (Safa and Solms, 2016; Abdillah et al., 2018; Esmaeelinezhad and Afrazeh, 2018; Islam et al., 2018; Afshar Jalili and Ghaleh, 2021; Jameel et al., 2021; Syed et al., 2021). Syed et al. (2021) applied the TPB to study the knowledge sharing behavior of academicians in HEIs. They mentioned that the behavioral intention and the actual sharing behavior both are important determinants of academicians’ knowledge sharing behavior. Hence, the research model in the present study is based on the TPB model. Based on the model, we have chosen “*attitude toward knowledge sharing*,” “*subjective norms*,” “*intention to share knowledge*,” and “*knowledge sharing behavior*” as the variables to study among the academicians.

In addition, apart from the dimensions of the TPB, additional variables are needed for understanding the academicians’ behavior in HEIs (Al-Kurdi et al., 2020). Such behavior must be investigated as a multi-dimensional construct so that a robust understanding can be achieved (Shaukat et al., 2023). Various studies have considered “motivation” as a crucial element for exhibiting knowledge sharing behavior (Bock et al., 2005; Lin, 2007; Razak et al., 2016). Furthermore, this dimension has been highly recommended to study in the context of academicians in HEIs (Asghar and Naveed, 2021; Jameel et al., 2021; Mutahar et al., 2022). Obrenovic et al. (2020) mentioned that it is important to understand what motivates or controls an individual to participate in knowledge sharing practice as human beings possess complex set of factors. Chedid et al. (2019) have suggested that HEIs should develop mechanisms based on motivational factors to promote knowledge sharing among its members. Therefore, the present study identifies the “*motivation to share knowledge*” as a key antecedent for knowledge sharing behavior. Additionally, facilitating conditions are equally important factors responsible for individuals to engage in some specific behavior. There are certain organizational factors which are outside the control of its members. These have major role in swaying employees to participate in knowledge sharing (Al-Kurdi et al., 2018). Even with strong intention, an individual will not be able to exhibit the behavior in presence of some hindrance. For example, these hindrances may be in the form of a lack of proper infrastructure, formal and informal meeting spaces, physical environment, and technology. These factors have been studied as “opportunities to share” by some previous studies (Sadiq Sohail and Daud, 2009; Lee and Hong, 2014; Islam et al., 2015). Such factors can play major role in enhancing the knowledge sharing behavior of the university teachers (Javaid et al., 2020; Syed et al., 2021). Hence, the present study identifies “*opportunities to share knowledge*” as a dimension critical to give direction to the knowledge sharing behavior of the academicians. The next sub-sections provide a description of these variables and the hypotheses framed based on the various possible relationships between them.

### 3.1. Attitude toward knowledge sharing and intention to share knowledge

The TPB has considered attitude as a key dimension to be studied for understanding an individual’s behavior (Ajzen, 1991; Chennamaneni et al., 2012; Kaba et al., 2023). Furthermore, many imperative studies have used this dimension to explain the knowledge sharing behavior of the academicians in HEIs (Fauzi et al., 2019; Al-Kurdi et al., 2020; Jameel et al., 2021; Syed et al., 2021; Chedid et al., 2022). Attitude can be defined as an individual’s positive or negative evaluation toward engaging in a specific behavior (Ajzen, 1991; Safa and Solms, 2016). It forms the basis of the individual’s past and present experiences resulting in his/her favoring or disfavoring a specific object or behavior (Shropshire et al., 2015; Afshar Jalili and Ghaleh, 2021). In context of knowledge sharing behavior, Bock et al. (2005) defined it as the degree of an individual’s positive feelings regarding sharing his/her knowledge. Mousa et al. (2019) defined attitude toward knowledge sharing as the “degree to which an individual has a favorable or bad KS assessment.” Jameel et al. (2021), in context of academicians, defined the concept as the favorable or unfavorable assessment of the academicians’ sharing behavior based on their sharing beliefs. Another important dimension identified by the TPB is an individual’s behavioral intention. Intention has been defined as an individual’s willingness to getting involved in a specific behavior (Ajzen, 1991). It shows the mental preparedness of a person to engage in certain actions at present or in the near future. In a knowledge sharing context, it refers to a person’s willingness to share knowledge with their colleagues, team, or organization (Safa and Solms, 2016). Knowledge sharing intention shows knowledge worker’s eagerness to get involved in knowledge sharing behavior (Wu and Zhu, 2012). Obrenovic et al. (2021) mentioned that employees showing stronger knowledge sharing intention gradually move to actual sharing action. In the context of academicians in HEIs, this is the academician’s readiness to share knowledge through research work, teaching, and conferences, etc. (Afshar Jalili and Ghaleh, 2021; Syed et al., 2021).

According to the TPB, attitudes toward a particular behavior and subjective norms have an impact on an individual’s intention to engage in the behavior (Ajzen, 1991; Al-Kurdi et al., 2020). In the context of knowledge sharing, attitudes are responsible for building an individual’s intention to exhibit knowledge sharing behavior (Bock et al., 2005; Lin, 2007; Alajmi, 2011; Skaik and Othman, 2015; Safa and Solms, 2016; Al-Kurdi et al., 2018). Various studies have found attitude as a strong predictor of behavioral intention in knowledge sharing (Chennamaneni et al., 2012; Abdillah et al., 2018; Obrenovic et al., 2021). In the context of academicians working in HEIs, Jameel et al. (2021) found a significant impact of academicians’ attitudes toward knowledge sharing on their intention to share knowledge. They have mentioned that academicians with a positive attitude for knowledge sharing will produce mature academicians willing to share their knowledge with others in the universities. Al-Kurdi et al. (2020) found academician’s attitudes toward knowledge sharing is a strong antecedent to the intention to share knowledge in HEIs. Further, the same result has been supported by some other studies (Chedid et al., 2019; Fauzi et al., 2019; Afshar Jalili and Ghaleh, 2021; Syed et al., 2021). Therefore, the researchers posit the first hypothesis as:

*H1*: An academician’s attitude toward knowledge sharing has a positive significant impact on their intention to share knowledge.

### 3.2. Subjective norms, attitude toward knowledge sharing, intention to share knowledge, and knowledge sharing behavior

Subjective norms can be defined as individual’s perceived social pressure to engage or not to engage in a specific behavior (Ajzen, 1991; Cheng et al., 2013). It refers to the belief regarding the acceptance or non-acceptance of a particular behavior (Negara et al., 2021). In the context of knowledge sharing, subjective norms provide an outline for employees to understand the behavior expected from them for sharing and learning. They also prescribe proper behavior to be exhibited in knowledge sharing (Obrenovic et al., 2021). These are the workplace rules and regulations that influence and assess employees’ behavioral adoption of knowledge sharing in the workplace (La Barbera and Ajzen, 2020). In the context of academicians working in HEIs, a subjective norm can be defined as the perception of other relevant stakeholders including colleagues, management, and the educational community at large that the academicians should share knowledge as an essential responsibility of knowledge management (Skaik and Othman, 2015; Safa and Solms, 2016; Fauzi et al., 2019). Such norms are the external compulsions that the academicians feel regarding getting involved in knowledge sharing as per the community’s expectations (Jameel et al., 2021).

According to the TPB, subjective norms are critical in influencing intention toward a specific behavior (Ajzen, 1991; Al-Kurdi et al., 2020). Numerous studies have found that an individual’s subjective norms have a positive significant impact on their intention to share knowledge (Skaik and Othman, 2015; Safa and Solms, 2016; Chedid et al., 2019; Obrenovic et al., 2020; Negara et al., 2021; Obrenovic et al., 2021; Kaba et al., 2023). In the context of academicians working in HEIs, numerous studies have reported subjective norms as a strong predictor of the intention to share knowledge (Chennamaneni et al., 2012; Fauzi et al., 2019; Al-Kurdi et al., 2020; Afshar Jalili and Ghaleh, 2021; Jameel et al., 2021). Afshar Jalili and Ghaleh (2021) found that knowledge sharing intentions among academicians can be derived from supportive subjective norms. Jameel et al. (2021) have also reported a positive and significant impact of subjective norms on the knowledge sharing intentions of academicians. Furthermore, when the individuals in an organization are motivated enough to follow group norms in comparison to their individual norms, their behavior is more likely to be a strong reflection of the group’s collective action instead of individual actions. In such situations, an individual’s subjective norms will possibly affect his/her behavioral intentions directly and indirectly through the attitude toward the behavior (Bock et al., 2005; Syed et al., 2021). Additionally, there are studies that show a positive significant impact of such norms on academicians’ actual knowledge sharing behavior (Al-Kurdi et al., 2020; Afshar Jalili and Ghaleh, 2021; Jameel et al., 2021; Syed et al., 2021). It is critical to test the relationship between academicians’ subjective norms and their actual knowledge sharing behavior. Hence, the study proposes the following hypotheses relating to subjective norms with the intention to share knowledge, attitude toward knowledge sharing, and knowledge sharing behavior:

*H2*: An academician’s subjective norms have a positive significant impact on their intention to share knowledge.

*H3*: An academician’s subjective norms have a positive significant impact on their attitude toward knowledge sharing.

*H4*: An academician’s subjective norms have a positive significant impact on their knowledge sharing behavior.

### 3.3. Intention to share knowledge and knowledge sharing behavior

Knowledge sharing behavior refers to a person’s actual response toward engaging in knowledge sharing, taking non-volitional controls under consideration (Ajzen, 1991; Safa and Solms, 2016). Some studies have defined this as the exchange of information taking place between the individuals to generate new knowledge, learn new skills, identify solutions to problems, and bring sustainable innovation to attain organizational goals effectively (Phong et al., 2018; Raza and Awang, 2020). Afshar Jalili and Ghaleh (2021) explained knowledge sharing behavior as “a set of discretionary activities of making personal valuable knowledge and experience actively available to others within an organization to foster organizational learning.” In the context of academicians working in HEIs, it refers to the actual actions taken by academicians in knowledge sharing through publications, conferences, community services, and professional activities (Massoudi et al., 2020; Syed et al., 2021).

According to the TPB, the stronger the intention to engage in a particular behavior, the more likely the individual will exhibit the actual behavior (Ajzen, 1991). It shows the link between beliefs and behavior, further indicating that behavior can be planned and is intentional (Wahyuni et al., 2020). Numerous studies have suggested that intention has a positive and significant effect on the knowledge sharing behavior of an individual (Lin and Lee, 2004; Wang and Noe, 2010; Alajmi, 2011; Obrenovic et al., 2020, 2021). In the context of academicians working in HEIs, their intention to share knowledge has been found to be critical for leading to actual knowledge sharing behavior (Fauzi et al., 2019; Afshar Jalili and Ghaleh, 2021; Jameel et al., 2021; Syed et al., 2021). Hence, the researchers propose the following hypothesis:

*H5*: The stronger an academician’s intention to share knowledge, the more likely he/she will engage in knowledge sharing behavior.

### 3.4. Motivation to share knowledge, attitude toward knowledge sharing, intention to share knowledge, and knowledge sharing behavior

The motivation to share knowledge has been defined as the driver responsible for an individual’s behavior toward sharing knowledge with others (Razak et al., 2016). Obrenovic et al. (2021) suggested that it should be the organization’s foremost priority to inspire their employees to engage in knowledge sharing, which if not attained may have an adverse impact on its sustainable goals. In context of HEIs, universities require their academic staff to be motivated to engage in knowledge sharing with a positive approach (Jameel et al., 2021). It is imperative to examine their motivations to share knowledge for implementing an effective knowledge management system (Asghar and Naveed, 2021). Chedid et al. (2022), in their study conducted among professors and researchers, reported that their motivation positively affects their knowledge sharing attitude. According to expectancy theory (Vroom, 1964), people are more inclined to perform an action if they perceive more positive outcomes associated with the specific action. A motive acts as a stimulus for a person to engage in a specific behavior (Safa and Solms, 2016). According to Ryan et al. (2010), an individual’s motivations arising from their needs and expectations may result in the showing of certain behavior. Various motivational factors like an employee’s promotion, reputation, and curiosity satisfaction can help in developing intentions to share knowledge among individuals (Safa and Solms, 2016). According to previous studies, willing individuals should be motivated to exhibit knowledge sharing behavior (Ziemba and Eisenbardt, 2014). Additionally, numerous studies reported a positive and significant relationship between several motivational factors (reciprocal benefits, knowledge self-efficacy, and enjoyment in helping others) and an employee’s attitude toward knowledge sharing as well as their intentions to share knowledge (Bock et al., 2005; Lin, 2007; Islam et al., 2018; Chedid et al., 2019; Javaid et al., 2020; Obrenovic et al., 2020). Furthermore, many researchers have identified that the extrinsic and intrinsic motivations of an individual to share knowledge have an impact on their actual knowledge sharing behavior (Bock et al., 2005; Negara et al., 2021; Obrenovic et al., 2021). Employees are more inclined toward knowledge sharing when some rewards are provided (Negara et al., 2021). Al-Kurdi et al. (2020) mentioned that it is not possible for any HEI to make it obligatory for the academicians to engage in actual knowledge sharing behavior, but such behavior can be nurtured through motivation. Therefore, the study posits the following three hypotheses:

*H6*: An academician with strong motivation to share knowledge will have a positive and significant attitude toward knowledge sharing.

*H7*: An academician with strong motivation to share knowledge will have a positive and significant intention to share knowledge.

*H8*: An academician with strong motivation to share knowledge will engage in knowledge sharing behavior.

### 3.5. Opportunities to share knowledge, attitude toward knowledge sharing, intention to share knowledge, and knowledge sharing behavior

Opportunities to share knowledge refers to the facilitating conditions allowing an individual to share knowledge with others (Sadiq Sohail and Daud, 2009). The facilitating conditions is a key organizational support that can trigger an employee’s readiness to share knowledge and innovative behavior (Kambey et al., 2018; Le and Lei, 2019; Obrenovic et al., 2020). In context of HEIs, Fauzi et al. (2019) emphasized providing facilitating conditions to academicians as it will help them to be in a positive state of mind for knowledge sharing practices. The HEIs must facilitate better services and the right circumstances for its academicians to inculcate knowledge sharing behavior among them (Massoudi et al., 2020; Jameel et al., 2021). Such facilitating conditions may be in the form of sound university infrastructure, formal and informal meeting places, the latest information and communication technology (ICT) support, and other resources (Sadiq Sohail and Daud, 2009). It is easy to influence or inculcate a behavior in the presence of sound facilitating conditions in the work environment. Some studies have found a significant relationship between such opportunities available to an individual within an organization and their knowledge sharing behavior (Radaelli et al., 2014; Sergeeva and Andreeva, 2016). Kumar et al. (2022) mentioned that facilitating conditions like IT support has a positive and significant influence on the knowledge sharing behavior of the employees. In context of HEIs, various studies have reported that the facilitating conditions have a key role in enhancing the knowledge sharing behavior of academicians (Sadiq Sohail and Daud, 2009; Fauzi et al., 2019; Javaid et al., 2020; Syed et al., 2021). Hence, taking all the behavioral elements including attitude, intention, and actual behavior into consideration, the study sets forth the following three hypotheses:

*H9*: The higher the number of opportunities for an academician to share knowledge in an organization, the more positive their attitude toward knowledge sharing will be.

*H10*: The higher the number of opportunities for an academician to share knowledge in an organization, the more positive their intention to share knowledge will be.

*H11*: An academician with a higher number of opportunities to share knowledge in an organization will engage in knowledge sharing behavior.

**Figure 1:**
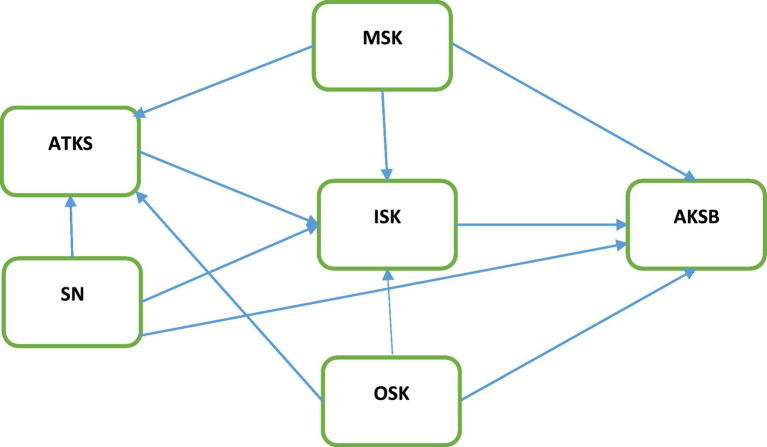
Hypothesized research model.

Based on the hypotheses posited by the researchers, Figure 1 shows the research model for the present study.

## 4. Research methodology

The researchers in the present study have proposed a conceptual model vis-a-vis the knowledge sharing behavior of the academicians. A 28-item research instrument comprising six factors has been adapted from the extant literature. The instrument comprised six items on attitude toward knowledge sharing (adapted from Bock et al., 2005; Sadiq Sohail and Daud, 2009), five and four items on subjective norms and the intention to share knowledge, respectively (adapted from Bock et al., 2005), four items each on the opportunities to share knowledge and motivation to share (adapted from Sadiq Sohail and Daud, 2009), and five items on knowledge sharing behavior (adapted from Cheng et al., 2009). The variable items were modified and rephrased by the researchers keeping in mind the respondents of higher educational institutions. Keeping in mind the cost-effectiveness and ease of collecting responses, a questionnaire was developed to collect data from the respondents (Bryman and Bell, 2014). The questionnaire comprised two sections; the initial one included demographic data like age, gender, designation, university/college type, and experience, and the second section comprised the abovementioned items measured on a 5-point Likert scale (1 = strongly disagree and 5 = strongly agree).

### 4.1. Sample and data collection

The objective of the study was to look into the knowledge sharing behavior of academic staff. The population of the present work is the academicians of higher academic institutions in India. Initially, faculty members of different colleges and universities of Delhi NCR were approached through email to fill out the survey but very few responses were recorded.

Universities and academic institutes are supposed to have a similar structure (Altbach, 2015). The response rate was meager, so to increase the responses, the researchers tried to reach out to faculty members of various institutes in India. The questionnaire link was shared through emails and social media groups. A snowball approach was also used to reach out to the maximum number of respondents. After all the efforts to get in touch with faculty members of different institutes and get their responses for the study, the researchers were only able to gather 112 filled responses for further analysis. However, this sample size is in line with the recommendations of Hair et al. (2010), who have suggested that SEM models can be estimated with a sample size as small as 100 if the number of factors and observed variables is not high and the model proposed is not complex.

Table 2 provides a summary of the demographic profile of the respondents. The population surveyed contained more male (71) than female (41) respondents. In terms of academic positions, assistant professors were the most common participants in the survey at around 80%, and the remaining 20% represented associate and full professors. Most of the respondents were from government-funded universities. In terms of experience, 40% of them had less than 5 years of experience, 37% had an experience of 5–10 years, while the remaining 23% had more than 10 years of experience.

**Table 2:** Demographic profiles of respondents.

	Frequency
Age	
21–35 Years	53
35–50 Years	54
Above 50	05
Gender	
Male	71
Female	41
Designation	
Assistant professor	91
Associate professor	10
Professor	11
University type	
Govt. funded	63
Private	49
Experience	
Less than 5 Years	45
5–10 Years	26
More than 10 years	41

## 5. Analysis and results

### 5.1. Confirmatory factor analysis

Confirmatory factor analysis (CFA) was employed in AMOS 24.0. The objectives for conducting CFA were to check the goodness of fit for the measurement model (DeVellis, 1991; Hair et al., 2010) and to assess the construct validity of the research instrument (Hair et al., 2010). CFA was performed on the six constructs. The item loadings for some items were low and hence these items were removed (ATKS2, ATKS5, ATKS6, KNOWS5, SN1, and SN2) (see Table 3). The model fit indices were acceptable (CMIN/df = 2.22; TLI = 0.91; CFI = 0.926; SRMR = 0.059; RMSEA = 0.0.085; Gerbing and Anderson, 1988; Hu and Bentler, 1999; Hair et al., 2010).

**Table 3:** Factor loadings.

			Estimate
ATKS3	<---	ATKS	0.890
ATKS1	<---	ATKS	0.848
ATKS4	<---	ATKS	0.841
KNOWS4	<---	KSB	0.735
KNOWS1	<---	KSB	0.807
KNOWS2	<---	KSB	0.861
KNOWS3	<---	KSB	0.837
OPTS3	<---	OSK	0.933
OPTS4	<---	OSK	0.849
OPTS2	<---	OSK	0.861
OPTS1	<---	OSK	0.847
INTSK4	<---	ISK	0.881
INTSK3	<---	ISK	0.855
INTSK2	<---	ISK	0.830
INTSK1	<---	ISK	0.809
SN4	<---	SN	0.846
SN5	<---	SN	0.823
SN3	<---	SN	0.762
MOTS4	<---	MSK	0.899
MOTS2	<---	MSK	0.863
MOTS3	<---	MSK	0.942

The values for average variance extracted (AVE) were also acceptable for all constructs (see Table 4) confirming convergent validity (Fornell and Larcker, 1981; Hair et al., 2010). The composite reliability (CR) values were also greater than 0.7 for all the constructs (Fornell and Larcker, 1981; Hair et al., 2010; Malhotra and Dash, 2011).

**Table 4:** Validity and reliability.

	CR	AVE	MSV	ATKS	KSB	OSK	ISK	SN	MSK
ATKS	0.895	0.739	0.482	**0.860**	-	-	-	-	-
KSB	0.885	0.658	0.442	0.665	**0.811**	-	-	-	-
OSK	0.928	0.763	0.183	0.293	0.272	**0.873**	-	-	-
ISK	0.919	0.740	0.482	0.695	0.651	0.205	**0.860**	-	-
SN	0.852	0.658	0.445	0.667	0.591	0.422	0.490	**0.811**	-
MSK	0.929	0.814	0.183	0.319	0.410	0.428	0.231	0.399	**0.902**

CR = Composite reliability; AVE = Average variance extract; MSV = Maximum shared variance. Diagonal value is the square root of AVE. The bold values are the square root of AVE determining the discriminant validity.

Discriminant validity was assessed by comparing the values of AVE and MSV (Fornell and Larcker, 1981; Hair et al., 2010). For all the constructs, the values of AVE were observed to be greater than the MSV values. The square root of the AVE values on the diagonal (highlighted in bold) were greater than the inter-construct correlations in their respective columns (see Table 4). The method of Heterotrait-monotrait ratio of correlations (HTMT) from Henseler et al. (2015) was also used to assess the discriminant validity. HTMT scores for all constructs were calculated using the plugin provided by Gaskin for AMOS 24.0. The HTMT scores for all the constructs were observed to be less than 0.85 (Table 5), thus confirming discriminant validity.

**Table 5:** Discriminant validity using HTMT scores.

	ATKS	KSB	OSK	ISK	SN	MSK
ATKS	-	-	-	-	-	-
KSB	0.660	-	-	-	-	-
OSK	0.305	0.280	-	-	-	-
ISK	0.721	0.681	0.244	-	-	-
SN	0.679	0.596	0.446	0.503	-	-
MSK	0.305	0.366	0.434	0.246	0.397	-

### 5.2. Common method bias

The common method bias was assessed using the common latent factor (CLF) method. The difference between standardized weights with CLF and without CLF was less than 0.200 for all the constructs, ensuring the non-existence of common method variance issues with the responses (Podsakoff et al., 2003; Serrano Archimi et al., 2018).

### 5.3. Structural model

Additionally, the structural model findings showed that the construct subjective norms (SN) significantly and positively affected the attitude towards knowledge sharing (ATKS; ß = 0.796; sig < 0.05) and actual knowledge sharing behavior (KSB; ß = 0.223; sig < 0.05). Hence, the hypotheses H3 and H4 were supported. However, the significant influence impact of SN on the intention to share knowledge (ISK) was not observed in the study findings (sig > 0.05). Thus, the hypothesis H2 was not supported.

Similarly, the hypotheses H9, H10, and H11 were not supported as the significant impact of the construct opportunities to share knowledge (OSK) was not observed on the constructs ATKS (sig > 0.05), ISK (sig > 0.05), and KSB (sig > 0.05). The construct motivation to share knowledge was observed to have a significant and positive impact on KSB (ß = 0.093; sig < 0.05) but not on the constructs ATKS (sig > 0.05) and ISK (sig > 0.05). Therefore, hypothesis H8 was supported but hypotheses H6 and H7 were not supported.

Furthermore, the study findings suggested a significant and positive impact (ß = 0.654; sig < 0.05) of ATKS on ISK. Also, the significant and positive impact of ISK on KBS was observed (ß = 0.344; sig < 0.05). Thus, the hypotheses H1 and H5 were supported. A summary of the results can be seen in Table 6.

**Table 6:** Results of hypotheses testing.

S.N.	Hypothesis Code	Path	Estimates	C.R	Result
1.	**H1**	**ATKS** → **ISK**	**0.654** *	**9.298**	**Supported**
2.	H2	SN → ISK	0.039	0.369	Not Supported
3	**H3**	**SN** → **ATKS**	**0.796** *	**8.396**	**Supported**
4.	**H4**	**SN** → **KSB**	**0.223** *	**3.698**	**Supported**
5.	**H5**	**ISK** → **KSB**	**0.344** *	**5.746**	**Supported**
6.	H6	MSK → ATKS	0.064	0.805	Not Supported
7.	H7	MSK → ISK	0.003	0.037	Not Supported
8.	**H8**	**MSK** → **KSB**	**0.093** *	**2.497**	**Supported**
9.	H9	OSK → ATKS	−0.011	−0.135	Not Supported
10.	H10	OSK → ISK	−0.007	−0.089	Not Supported
11.	H11	OSK → KSB	−0.042	−0.584	Not Supported

* Significant < 0.05.

The bold values indicate supported hypothesis.

Model fit indices for the final structural model were within the acceptable range (CMIN/df = 2.056; CFI = 0.946; TLI = 0.934; SRMR = 0.056; RMSEA = 0.079).

## 6. Findings and discussion

A review of extant literature supports the robustness of the TPB in predicting behavioral intentions (Razak et al., 2016). To improve the predictive power of the knowledge sharing behavior model, the researchers in the present study included two other important variables, namely, *opportunities to share knowledge* and *motivation to share knowledge*, along with the variables of the TPB. Several researchers have claimed that these two variables play an important role in predicting knowledge sharing behavior (Bock et al., 2005; Sadiq Sohail and Daud, 2009; Wang and Noe, 2010; Razak et al., 2016; Safa and Solms, 2016). Thus, in the present study, two additional variables, MOTS and OPTS, were introduced with other factors “*attitude toward knowledge sharing*,” “*subjective norms*,” “*intention to share knowledge*,” and “*knowledge sharing behavior*” to better explain the knowledge sharing behavior of academicians.

The results of the structural model signify that intention to share knowledge, motivation to share knowledge, and subjective norms have a strong effect on the actual knowledge sharing behavior of the academicians. The opportunity to share knowledge had no effect on the actual knowledge sharing behavior. The analysis also suggested that subjective norms positively impacted the attitude towards knowledge sharing and knowledge sharing behavior but interestingly had no impact on the intention to share knowledge of the academicians. The study findings related to SN are in line with several studies (Fauzi et al., 2019; Al-Kurdi et al., 2020; Afshar Jalili and Ghaleh, 2021; Jameel et al., 2021). Attitude to share knowledge had a significant positive impact on intentions to share knowledge, with the findings being in line with the results of Bock et al. (2005), Lin (2007), Alajmi (2011), Chennamaneni et al. (2012), Skaik and Othman (2015), Safa and Solms (2016), Abdillah et al. (2018) and Obrenovic et al. (2021). The findings of the studies by Lin and Lee (2004), Wang and Noe (2010), Alajmi (2011), Fauzi et al. (2019), Afshar Jalili and Ghaleh (2021), Jameel et al. (2021), and Syed et al. (2021), support the findings of the present study in that the intention to share knowledge has a significant and positive influence on knowledge sharing behavior. Interestingly, the results of the present study did not find motivation to share to knowledge determined attitude and intention but positively impacted knowledge sharing behavior, which earlier studies (Islam et al., 2018; Chedid et al., 2019; Javaid et al., 2020; Obrenovic et al., 2020) have suggested to be an important factor in knowledge sharing behavior. On the other hand, the opportunity to share knowledge had no impact on attitude, intention, or the actual knowledge sharing behavior of the academicians, and these findings are not in line with the findings of Bock et al. (2005) and Sadiq Sohail and Daud (2009).

The findings of the present research provide theoretical as well as practical suggestions in determining and explaining the knowledge sharing behavior of academicians. The theoretical contribution of the present research is four-fold. First, the researchers in the present work have offered novelty by expanding the extant research by demarcating factors that affect the knowledge sharing behavior of academicians. Second, the present study offers an integrated and extended TPB theoretical model, augmenting it with constructs like motivation and opportunity to share knowledge adapted from related research findings. Third, the researchers in the present study have tried to present a shorter and more reliable scale which can be used to assess the behavioral intentions of academicians to share knowledge. Fourth, the findings of the study resulted in an intention-based knowledge sharing behavior model which connotes its robustness. Academicians in developed nations differ from those in emerging countries where the infrastructure and facilities are not so supportive. Thus, the present study offers insight into knowledge sharing behavior of the academicians from the perspective of academicians from a developing country like India.

Knowing the relevance of knowledge sharing behavior among faculty members in any institute, the administration always ponders to develop the culture among the faculty members. Besides the theoretical contributions, the findings of the present study offer practical implications for the administrators of universities. From an administrative perspective, the findings suggest that subjective norms, attitude to sharing knowledge, and motivation to share knowledge are strong predictors of the actual knowledge sharing behavior of the academic community. Thus, it might be recommended that the administration motivates academicians to build positive attitudes towards the sharing of knowledge, for example by offering different types of incentives and rewards.

## 7. Limitations and directions for future research

Like any piece of research, the present study has limitations that should be considered carefully. First, the researchers used a researcher-controlled sample to generate data for this study, and the small sample size might restrict the generalizability of the findings. Future researchers may validate the results by relying on a broader and a more representative probability-based sample. The second limitation is based on the factors used in the study. The present study has mainly focused on a few factors and therefore some relevant factors determining knowledge sharing behavior might have been overlooked. Future researchers may take into account other important factors, like organizational culture and leadership. These factors might have an effect on knowledge sharing behavior. Third, the respondents in the present study were only academicians, and future studies may broaden the scope by analyzing other types of sectors of the economy.

## Data availability statement

The data supporting the conclusions of this article may be provided on request. Requests to access the data should be directed to the corresponding author.

## Author contributions

All authors listed have made a substantial, direct, and intellectual contribution to the work and approved it for publication.

## Conflict of interest

The authors declare that the research was conducted in the absence of any commercial or financial relationships that could be construed as a potential conflict of interest.

## Publisher’s note

All claims expressed in this article are solely those of the authors and do not necessarily represent those of their affiliated organizations, or those of the publisher, the editors and the reviewers. Any product that may be evaluated in this article, or claim that may be made by its manufacturer, is not guaranteed or endorsed by the publisher.
